# A Cross-Lingual Similarity Measure for Detecting Biomedical Term Translations

**DOI:** 10.1371/journal.pone.0126196

**Published:** 2015-06-01

**Authors:** Danushka Bollegala, Georgios Kontonatsios, Sophia Ananiadou

**Affiliations:** 1 Department of Computer Science, University of Liverpool, United Kingdom; 2 School of Computer Science, University of Manchester, Manchester, United Kingdom; 3 National Centre for Text Mining, University of Manchester, Manchester, United Kingdom; University of Illinois-Chicago, UNITED STATES

## Abstract

Bilingual dictionaries for technical terms such as biomedical terms are an important resource for machine translation systems as well as for humans who would like to understand a concept described in a foreign language. Often a biomedical term is first proposed in English and later it is manually translated to other languages. Despite the fact that there are large monolingual lexicons of biomedical terms, only a fraction of those term lexicons are translated to other languages. Manually compiling large-scale bilingual dictionaries for technical domains is a challenging task because it is difficult to find a sufficiently large number of bilingual experts. We propose a cross-lingual similarity measure for detecting most similar translation candidates for a biomedical term specified in one language (source) from another language (target). Specifically, a biomedical term in a language is represented using two types of features: (a) *intrinsic features* that consist of character n-grams extracted from the term under consideration, and (b) *extrinsic features* that consist of unigrams and bigrams extracted from the contextual windows surrounding the term under consideration. We propose a cross-lingual similarity measure using each of those feature types. First, to reduce the dimensionality of the feature space in each language, we propose *prototype vector projection* (PVP)—a non-negative lower-dimensional vector projection method. Second, we propose a method to learn a mapping between the feature spaces in the source and target language using *partial least squares regression* (PLSR). The proposed method requires only a small number of training instances to learn a cross-lingual similarity measure. The proposed PVP method outperforms popular dimensionality reduction methods such as the singular value decomposition (SVD) and non-negative matrix factorization (NMF) in a nearest neighbor prediction task. Moreover, our experimental results covering several language pairs such as English–French, English–Spanish, English–Greek, and English–Japanese show that the proposed method outperforms several other feature projection methods in biomedical term translation prediction tasks.

## Introduction

Technical terms are coined in many domain on a daily basis. In specialized domains such as medicine, technical terms are often first proposed in English and later translated into other languages. Finding proper translations for technical terms is an important factor that expedites the technical knowledge across languages. Therefore, bilingual dictionaries for technical terms play an important role in both manual [1] and machine translation [2] approaches. Unfortunately, only a small fraction of the technical terms proposed in English are translated into other languages, which is problematic for machine translation systems that require bilingual term lexicons. For example, Unified Medical Language System (UMLS) Metathesaurus (http://nlm.nih.gov/research/umls), one of the comprehensive multilingual medical resources covering 21 languages, contains 75.1% English terms, 9.99% Spanish terms, 2.22% Japanese terms, and 1.82% French terms. The unbalanced representation of languages other than English in UMLS demonstrates the severity of the problem of technical term translation.

Manual translation of technical terms is a challenging task due to several reasons. First, it is difficult to find bilingual experts in highly technical domains who are willing to manually translate technical terms from one language to another. Second, domains such as medicine are so vast that it is difficult to find enough bilingual experts to cover all sub-domains within a domain. Therefore, technical terms in some sub-domain within a large domain might not be sufficiently translated to other languages. Third, it is often difficult for humans to construct new translations from scratch although it is relatively easier to determine whether two words are suitable translations of each other. As a practical solution to this problem, we could assist human translators by providing a short list of translation candidates for a given technical term, thereby minimizing their effort to go through large term lexicons. Dictionaries and bilingual terminological resources are quickly out-of-date by the moment they are compiled due to the number of publications appearing in specialized domains such as medicine. Therefore, automatic methods for detecting translations for technical terms are important from the perspective of update and maintenance.

In this paper, we model the problem of detecting translations of technical terms as a cross-lingual similarity measurement task [3]. Specifically, given a term *w*_𝓢_ in a source language 𝓢, and a term *w*_𝓣_ in a target language 𝓣, we propose a cross-lingual similarity measure, sim(*w*_𝓢_, *w*_𝓣_), that indicates the semantic similarity between the two terms *w*_𝓢_ and *w*_𝓣_. Throughout this paper, we use *term* to refer to single words or multi-word expressions that are used as technical terms in a particular field. We represent each term *w* in a language ℒ (which could be either 𝓢 or 𝓣) using two types of features: (a) character n-grams extracted from *w*, and (b) contextual lexical features such as unigrams and bigrams extracted from the occurrences of *w* in a corpus. Character n-grams can be considered as *intrinsic* features that represent various properties of the word under consideration such as syllables, inflections, and its etymological structure. For example, the three kanji character sequence 症候群 (*sho-kou-gun*) is often translated to English as *syndrome*. Therefore, any medical term that contains these three character sequence can be translated as some syndrome with a high probability. Character n-gram features are particularly useful for translating technical terms that follow the *compositionality principle* (i.e. the meaning of a multi-word term is composed by the meanings of its constituent words). On the other hand, contextual features can be considered as *extrinsic* features that indicate the words frequently associated with the word under consideration. For example, names of diseases associated with a particular body organ are likely to appear within close proximity of the mentions of that organ in a text. Therefore, the two feature types are complementary in the sense that character n-grams are extracted from the terms themselves, whereas contextual features are extracted using an external corpus. We experimentally evaluate the effect of those two feature spaces for computing cross-lingual similarity.

Although two terms *w*_𝓢_ and *w*_𝓣_ could be represented using various features as described in the previous paragraph, the two feature representations created for different languages will have low overlap in practice. For example, consider the English source and Japanese target language setting. The two languages use different alphabets. Therefore, character n-gram features extracted for terms in English and Japanese languages will have no overlap. Likewise, contextual features are also less likely to overlap. Therefore, popular similarity measures such as the cosine similarity between two feature vectors representing the two terms would often return zero similarity scores. To overcome this problem, we first learn a projection from the source language feature space to the target language feature space. Specifically, we model the projection learning problem as a multi-variate regression problem and use Partial Least Squares Regression (PLSR) [4] to learn a projection *M* from the source to the target language feature space. Next, we project the feature vector ***w***_**𝓢**_ for the source term *w*_𝓢_ to the target language feature space using the learnt projection. Let us denote the projection operation by *M*(***w***_**𝓢**_). We train a binary Random Forest (RF) classifier [5] to classify word pairs (*w*_𝓢_, *w*_𝓣_) depending on whether *w*_𝓣_ is the correct translation of *w*_𝓢_. We represent a word pair (*w*_𝓢_, *w*_𝓣_) by a feature vector [***w***_**𝓢**_;***w***_**𝓣**_;*M*(***w***_**𝓢**_)], where we concatenate the three vectors ***w***_**𝓢**_, ***w***_**𝓣**_, and *M*(***w***_**𝓢**_). Finally, the similarity sim(*w*_𝓢_, *w*_𝓣_) between the source term *w*_𝓢_ and each of the target terms *w*_𝓣_ is computed as the class conditional probability returned by the random forest classifier expressing the likelihood of *w*_𝓣_ being the correct translation of *w*_𝓢_. We rank the target language terms *w*_𝓣_ in the descending order of their similarity scores to the source term *w*_𝓢_, and display the top-ranked terms as the potential translation candidates for *w*_𝓢_. A human annotator can then pick the best translation(s) from this short-listed candidates set.

An important challenge when learning a projection between high dimensional feature spaces such as character n-grams or contextual features is that the number of parameters in the projection model tends to be large. In practice, to accurately learn this large number of parameters in a feature projection model we would require a large training dataset. However, this could be a problem in bilingual term translation because, the number of terms that are already translated between two languages are likely to be small, which prompts for automatic methods for detecting term translations in the first place. Therefore, it is desirable to design automatic term translation methods that can learn from a small number of training instances. For this purpose, we propose Prototype Vector Projection (PVP), a dimensionality reduction method to first project the source and target feature vectors to a lower-dimensional space. The Prototype Vector Projection first selects a set of *d* *prototype vectors* from the feature vectors (*n* > > *d* in total) representing terms in a language. We propose a method for selecting prototype vectors based on the number of non-zero elements in feature vectors. Each feature vector is then projected to a *d*-dimensional space by taking the inner product with each of the prototype vectors. We finally learn a projection from the source to the target languages in this lower dimensional spaces using partial least squares regression (PLSR). PVP has several attractive properties such as: (a) the projections are always non-negative, given that the feature vectors are non-negative, (b) it does not require computationally expensive operations such as the Singular Value Decomposition (SVD), and (c) the basis vectors (prototypes) are always actual data points, which make the interpretation of the lower-dimensional space easier.

We evaluate the proposed cross-lingual similarity measure for its ability to correctly identify the translations for biomedical terms using four datasets: English-French terms, English-Spanish terms, English-Greek terms, and English-Japanese terms. We use Precision@R defined as the ratio between the total number of correct translations ranked among the top R candidates to the rank R as the evaluation measure. Precision@R is a popular evaluation measure in information retrieval where it is considered desirable to rank relevant search results among the top ranked documents to a query. We compare PVP against several widely used dimensionality reduction methods such as SVD or non-negative matrix factorization (NMF) using two synthetic datasets. PVP outperforms those methods in its ability to preserve the relative ranking of the nearest neighbors in the original space in the embedded space. Moreover, our experimental results in a cross-lingual biomedical term translation detection task show that PVP outperforms SVD and NMF on both character n-gram features as well as contextual features for different language pairs.

## Related Work

Measuring similarity between words and texts [6–8] is a fundamental task in Natural Language Processing (NLP) that is required for numerous other tasks such as document/word clustering [9], information retrieval [10–12], query suggestion [13], and word sense disambiguation [14]. A typical approach for measuring the similarity between two words is to first represent each word using the distribution of other words that co-occur with it in a corpus. The co-occurrences could be weighted using a suitable word association measure such as the pointwise mutual information. Next, the similarity between two words is computed using a linear algebraic operation over the vectors such as their inner-product. Although this procedure for measuring similarity between words has been highly successful for measuring the similarity between words in the same language, it fails in the cross-lingual setting because, the distributional vectors that represent words in different languages have minimal or no overlap [15]. Therefore, we must project the feature spaces for each language into some common sub-space before we can compute the similarity between words selected from two different languages.

In cross-lingual latent semantic indexing [16], Singular Value Decomposition (SVD) is performed on a set of parallel texts such as the Hansard collection, which is written both in English and French. Documents are represented using a matrix where each row corresponds to a pair of parallel documents, and each column represents a word in source or target languages. The SVD step reduces the number of columns, thereby embedding words that are distributed similarly in the two languages in the same latent dimension. However, this method requires a collection of parallel texts, which is problematic in our setting of biomedical term translation using monolingual lexicons. The manual effort required to translate biomedical documents such as scientific papers to create parallel corpora is significantly larger compared to manually translating a set of biomedical terms. Considering that most machine translation methods require either parallel or comparable corpora to train from, this requirement rules out the applicability of such methods to the problem-setting we consider in this paper.

Neologisms are often created by combining one or more existing terms. The principal of compositionally (also known as the Frege’s principle) states that the meaning of a multiword expression such as a technical term can be composed using the meanings of its constituent parts [17]. Compositional approaches for term translations exploit this property by individually translating each component of a term given in a source language using a bilingual dictionary, and subsequently scoring each generated target language translation candidate using some goodness measure. In lexical approaches [18–21], an input source term is segmented into basic translation units, whereas sub-lexical approaches [22] perform further segmentation into morphemes. Numerous goodness scores for evaluating translation candidates have been proposed in the literature such as, the frequency in a target language corpus [19, 20], or the contextual similarity with the source term [21]. Unfortunately, the performance of compositional approaches for term translation depends heavily on the coverage of the bilingual seed dictionary that is used for translating lexical or sub-lexical basic units. For example, it has been reported that as much as 30% of incorrectly translated terms by such compositional methods are due to the poor coverage of the seed dictionary [22].

Kontonatsios et al. [23] proposed a binary classification approach for detecting biomedical term translations. They represent biomedical terms using character n-grams extracted from the terms and train a binary random forest classifier [5]. A manually annotated set of term pairs selected from the source and the target languages is used as the positive training instances, whereas the negative training instances are created by randomly pairing two terms from each language. They evaluate the performance of their method using two language pairs: English-French and English-Chinese. They use a balanced dataset that contains equal numbers of positive and negative (i.e. correct vs. incorrect translations) word pairs, and use classification accuracy as the performance measure. However, in practice, we have only a handful of correct translations for a biomedical term although there will be many incorrect translations. Therefore, the balanced dataset approach does not reflect the true performance of a translation detection method when used in real-world systems such as, to suggest translation candidates to human annotators. Moreover, they do not consider contextual features, nor do they learn a mapping between the feature spaces corresponding to the two languages.

A bilingual dictionary-based approach for translating feature spaces between two languages to improve the accuracy of the cross-lingual similarity measurement is proposed by Kontonatsios et al. in [24]. They use both character n-grams as well as contextual features to represent terms in a language. Contextual features are translated by looking them up in a bilingual dictionary. Unlike technical terms that have unambiguous translations, contextual features consist of common words that can be ambiguous and are difficult to translate without considering their context. Therefore, using a bilingual dictionary to translate contextual features would introduce additional noise to the term translation detection process. Moreover, the requirement for an additional bilingual dictionary besides the train word pairs could be problematic for resource poor languages.

Measuring cross-lingual lexical similarity has been studied in the cross-lingual information retrieval and the machine translation communities [25, 26]. One line of approaches is to create cross-lingual topic models using parallel or comparable corpora such that words that express similar meaning in different languages are allocated to the same topic. Next, the similarity between two documents or words in the two languages can be computed using the posterior probabilities over the learnt topics. Numerous techniques can be used to learn cross-lingual topic models such as non-negative matrix factorization [27, 28], probabilistic principal component analysis [29], matching canonical correlation analysis [30], and multilingual probabilistic topic models [31, 32].

Our work in this paper is different from all of the above-mentioned prior work in several aspects. Firstly, we do not assume the availability of parallel or comparable corpora for the languages between which we must identify term translations. Secondly, we do not assume the availability of a bilingual dictionary that contains translations for non-technical terms such as common words as required by Kontonatsios et al. [24] for translating feature spaces. We assume that we are given two lists of terms (lexicons) extracted for each language using some method. Such lists are often created manually for indexing and categorization purposes. It is also possible to extract technical terms using automatic methods [1, 18] such as the C-value method [33]. Thirdly, we do not assume any properties of the term extraction method being used for creating term lists. Therefore, our proposed method can be used in principle to align term lists created using any automatic or manual methods.

## Cross-lingual Term Similarity

### Problem Definition

Given a list of terms *Q*_𝓢_ in a source language 𝓢, and a list of terms *Q*_𝓣_ in a target language 𝓣, we consider the problem of finding one or more translations for each term *w*_𝓢_ ∈ *Q*_𝓢_ from the target term list *Q*_𝓣_. We assume the availability of a small seed list of term pairs {(*w*_𝓢_, *w*_𝓣_)} for learning a cross-lingual similarity measure, sim(*w*_𝓢_, *w*_𝓣_), that indicates the degree of similarity between *w*_𝓢_ and *w*_𝓣_.

The trained cross-lingual similarity measure is used as follows. For each source term *w*_𝓢_, we measure its similarity to each of the target terms *w*_𝓣_, and rank the target terms in the descending order of their similarity scores sim(*w*_𝓢_, *w*_𝓣_). We display the top *N* ranked candidates to a human annotator to assist the process of finding term translations.

Two main approaches can be used to obtain a set of train term pairs {(*w*_𝓢_, *w*_𝓣_)} required by the proposed method. First, we can use monolingual term extraction methods [18, 34] to extract two separate term lists for the source and target languages. For example, Xu et al. [35] proposed a language-independent method for extracting large collections of medical terms from semi-structured information sources on the Web. Next, a human annotator can manually align some of the words in the source term lexicon to their correct translations in the target term lexicon. Obviously, there is no guarantee that all the terms found in the source term lexicon will have a corresponding translation in the target language lexicon. However, it is relatively easier for human annotators to find a translation for a given source language term from a list of candidate target language terms than to come up with a translation by themselves, without having access to a target language term lexicon.

An alternative second approach would be to use bilingual lexicon extraction methods [36–38]. These methods rely on comparable corpora such as Wikipedia articles written in different languages on the same topic or news articles published in different language on the same news event to measure similarity between terms written in different languages. One important problem that needs to be addressed is the ambiguity of translations because a single source term can be translated to multiple different target language terms. For example, Bouamor et al. [37] use WordNet to disambiguate contextual vectors when measuring cross-lingual distributional similarity. Compared to the first approach, which requires a human to manually align two monolingual term lexicons, the second approach is attractive because it enables us to obtain a large bilingual term lexicon for a relatively lower cost. If we would like to further improve the quality of the train term pairs, then we can perform manual filtering on top of the automatically extracted bilingual term lexicon.

Extracting train term pairs is beyond the scope of the current paper. In the subsequent discussion, we assume the availability of such a train dataset, without attempting to extract it from comparable corpora. More importantly, our proposed method for learning a cross-lingual similarity measure does not assume any specific properties for the methods used for extracting train term pairs. Therefore, in principle the proposed cross-lingual similarity measure can be used to align term lists extracted from any term extraction method.

### Monolingual Feature Vector Construction

The first step in our proposed method is to represent a term using a feature vector extracted from one of the source or the target languages. We refer to this step as the *monolingual feature vector construction* because the features we extract to represent a term *w* in a language ℒ consists only of features in language ℒ. Specifically, we consider two types of features.

- **Character n-gram Features**:
  We extract character n-grams for n = 2, 3, 4, and 5 from the term *w* as features. We then count the frequency of each n-gram extracted from all the terms in the training data, and select the most frequent n-grams. In our experiments, we consider several n-gram feature spaces consisting of different numbers of n-gram features. Character n-grams can express different semantic information in different languages. For example, in phonetic languages such as English, French, Spanish, or Greek, character n-grams can capture the inflections or etymological components in the terms. On the other hand, in pictorial languages such as Japanese or Chinese, a single *kanji* character encodes rich semantics regarding the term such as its semantic compositionality. We refer character n-grams as *intrinsic* features in this paper because they are extracted from the terms only, without requiring any external resources such as corpora, other than the term lexicons for the source and the target languages. We set binary feature values for the character n-grams extracted from a term. For example, consider the English term *catecholamine*. Some of the character n-grams extracted from this term are (*n* = 2) *ca, at, te*, (*n* = 3) *cat, ate, tec*, (*n* = 4) *cate, atec, tech*, and (*n* = 5) *catec, atech, techo*.
- **Contextual Features**:
  Although character n-grams are useful as intrinsic features for term alignment, they do not provide any information as to how those terms are used in a particular context. The distributional hypothesis, often succinctly expressed using the memorable quote from Firth [39] – “you shall know a word by the company it keeps”, states that we can obtain useful insights into the semantics of a term by looking into the contexts in which that term appear in a corpus. We consider all occurrences of a term *w* in a corpus, and extract unigrams and bigrams of tokens that appear within a 5-token window surrounding the term *w*. Specifically, the two tokens preceding and the two tokens succeeding a term are considered as its contextual window. In our experiments, we used the freely available Wikipedia (https://www.wikipedia.org/) corpus for extracting contextual features.
  For each extracted contextual feature we count the number of different train terms from which it is extracted. We then select the most frequent contextual features for representing terms. In our experiments we select the most frequent 10,000 contextual features from the source and the target languages. We set the value of a contextual feature *c* extracted for a term *w* to the positive pointwise mutual information (PPMI) [40]. PPMI is computed as
  $$\begin{array}{c} \mathrm{PPMI}\left(c,w\right)=\max\left(0,\log(\frac{p(c,w)}{p(c)p(w)})\right), \end{array}$$ (1)
  where *p*(*c*), *p*(*w*) respectively denote the marginal probabilities of the contextual feature *c* and the term *w*, and *p*(*c*, *w*) denotes their joint probability. Note that the logarithmic term in Eq 1 can become negative if the joint probability of *c* and *w* (i.e. *p*(*c*, *w*)) is lesser than what would be expected if *c* and *w* were mutually exclusive (i.e. *p*(*c*)*p*(*w*)). This negative association of words is a result of incorrect probability estimates and is removed by setting to zero in the PPMI formula. We estimate those probabilities using the number of contexts in which *c* and *w* co-occur in the corpus as follows
  $$\begin{array}{ccc} p(c) & = & \frac{\text{no.}\;\text{of}\;\text{contexts}\;\text{in}\;\text{which}\;c\;\text{occurs}}{\text{total}\;\text{no.}\;\text{of}\;\text{contexts}},\\ p(w) & = & \frac{\text{no.}\;\text{of}\;\text{contexts}\;\text{in}\;\text{which}\;w\;\text{occurs}}{\text{total}\;\text{no.}\;\text{of}\;\text{contexts}},\\ p(c,w) & = & \frac{\text{no.}\;\text{of}\;\text{contexts}\;\text{in}\;\text{which}\;c\;\text{and}\;w\;\text{co-occur}}{\text{total}\;\text{no.}\;\text{of}\;\text{contexts}}. \end{array}$$
  We refer to contextual features of a term as *extrinsic* features because they are extracted not from the term under consideration, but from the contexts in which that term occur. Character n-grams and contextual features are by design mutually exclusive and capture different properties of the terms.

### Prototype Vector Projection

The character n-gram feature space and contextual feature space in practice consist of numerous features resulting in very high dimensional feature spaces. For example, character n-grams feature space can exponentially grow with the length of the n-gram. This is problematic when learning a mapping from the source to the target language feature spaces because, a projection model could easily overfit with a large number of parameters. For example, if the source and the target feature spaces are *n*-dimensional, we require an *n* × *n* projection matrix to project source language feature vectors to the target language feature space in order to measure cross-lingual term similarities. Although such a projection matrix must be symmetric, hence requiring us to estimate only a half of the required total number of *n* ^2^ parameters, it is still a challenging parameter estimation problem due to the limited availability of training data in bilingual term lexicons. Moreover, the feature vectors representing terms in a language will be highly sparse because only a handful of character n-gram and contextual features will represent a particular term. A popular solution proposed in the prior work on document similarity measurement to overcome this data sparseness problem in high dimensional feature spaces is to perform dimensionality reduction as a pre-processing step [40].

We propose Prototype Vector Projection (PVP), a dimensionality reduction method that uses a subset of the feature vectors as the basis vectors, and project all feature vectors spanned by these basis vectors. We refer to the set of basis vectors selected as the *prototype vectors*. Given a set of *n* terms represented by *m*-dimensional feature vectors, PVP selects a subset of *d* < *n* vectors from this set as the prototype vectors. Pseudo code for PVP is shown in Table 1 Algorithm 1. First, we compute the centroid vector of the given set of feature vectors (Lines 2–4). Next, we rank the feature vectors by the number of non-zero elements that exist in the elementwise product between the centroid vector and each of the feature vectors. Elementwise product between two *m*-dimensional vectors (*x*_1_, *x*_2_, …, *x*_*m*_)^⊤^ and (*y*_1_, *y*_2_, …, *y*_*m*_)^⊤^ is defined as the *m*-dimensional vector (*x*_1_ *y*_1_, *x*_2_ *y*_2_, …, *x*_*m*_ *y*_*m*_)^⊤^. As we show later in Proposition 1, the score *s*(**x**,**c**(𝓧)) indicates the likelihood that we would obtain dense vectors after the projection when we use a feature vector as a prototype vector. Ideally, we would like to obtain dense vectors in the embedded space compared to that in the original space because, this would reduce the number of zero cosine similarity scores, thereby enabling us to measure similarity between terms more accurately. We select the feature vector with the maximum score as a prototype vector and remove it from the current set of feature vectors. We repeat this process until we are left with exactly *d* number of prototype vectors. By removing the selected prototype vectors from the set of feature vectors that we use for computing the centroid in each iteration we increase the diversity of the prototype vectors. Finally, we perform the Gram-Schmidt orthonormalization on the selected set of prototype vectors to create an orthonormal set of basis vectors [41]. Gram-Schmidt process produces a basis of orthonormal unit vectors from a given set of independent vectors. We project each feature vector to the *d*-dimensional space spanned by the prototype vectors by computing the inner-product of a feature vector with each of the prototype vectors. PVP is similar to random projection methods such as the locality sensitive hashing (LSH) [42, 43] in the sense that all feature vectors are projected to a lower dimensional space by taking the inner product with a set of vectors.

**Table 1:** **Algorithm 1** Prototype Vector Projection.

**Input**: A set of *n* of *m* dimensional feature vectors {**x**_1_, …,**x**_*n*_}, the number of prototype vectors *d*.
**Output**: The set $\left\{{\tilde{\text{x}}}_{1},\ldots ,{\tilde{\text{x}}}_{n}\right\}$ of projected vectors in the *d*-dimensional space.
1: Set of prototype vectors 𝓟 = {}
2: 𝓧 = {***x***_1_, …,***x***_*n*_}
3: **for** *i* = 1 **to** *d* **do**
4: Compute the centroid vector ***c***(𝓧) of the set of vectors in 𝓧 as follows $$\begin{array}{c} c\left(𝓧\right)=\frac{1}{n}\sum_{x\in 𝓧}x \end{array}$$
5: Compute the score, *s*(***x***,***c***(𝓧)), for each ***x*** ∈ 𝓧 by $\sum_{j=1}^{m}\text{I}(x_{j}c_{j}\neq 0)$. Here, *x*_*j*_ and *c*_*j*_ are the *j*-th dimensions of ***x*** and ***c***(𝓧), and I is the indicator function defined as follows $$\begin{array}{c} I\left(\theta \right)=\{\begin{array}{cc} 1 & \theta =\text{True}\\ 0 & \text{otherwise} \end{array} \end{array}$$
6: Select the vector x* ∈ 𝓧 with the highest score. i.e. ${\text{x}}^{*}=\underset{\text{x}\in 𝓧}{\text{argmax}}s(\text{x},\text{c}(𝓧))$.
7: 𝓟 = 𝓟∪{x*}
8: 𝓧 = 𝓧 {x*}
9: **end for**
10: Perform Gram-Schmidt orthonormalization on 𝓟 to obtain an orthonormal set of unit-length basis vectors {***p***_1_, …, ***p***_*d*_}.
11: **for** ***x***_*i*_ ∈ {***x***_1_, …,***x***_*n*_} **do**
12: $\tilde{x_{i}}={\left[x^{⊤}p_{1},\ldots ,x^{⊤}p_{d}\right]}^{⊤}$, Here, ***p***_1_, …,***p***_*d*_ are the prototype vectors computed and ordered following the procedure in Lines 3–10.
13: **end for**
14: **return** $\left\{{\tilde{\text{x}}}_{1},\ldots ,{\tilde{\text{x}}}_{n}\right\}$

**Proposition 1**. *The average number of non-zero elements in the elementwise product between a prototype vector **y** and a feature vector **x**_i_ ∈ {**x**_1_, …,**x**_n_} is given by the number of non-zero elements in the elementwise product between **y** and the centroid vector of {**x**_1_, …,**x**_n_}.*

*Proof*. Recall that the elements in the feature vectors ***x***_1_, …,***x***_*n*_ are non-negative. Let us define *s*(***x***_*i*_,***y***) as the number of non-zero elements in the elementwise product between **x**_*i*_ and **y**. It is given by,

$$\begin{array}{c} s\left(x_{i},y\right)=\sum_{j=1}^{m}I\left(y_{j}x_{ij}\neq 0\right). \end{array}$$

Here, *x*_*ij*_ indicates the *j*-th dimension of the vector ***x***_*i*_. Then, the average number of non-zero elements in the elementwise products between **y** and each of the feature vectors in {***x***_1_, …,***x***_*n*_} is given by,

$$\begin{array}{ccc} \frac{1}{n}\sum_{i=1}^{n}s\left(x_{i},y\right) & = & \frac{1}{n}\sum_{i=1}^{n}\sum_{j=1}^{m}I\left(y_{j}x_{ij}\neq 0\right)\\ & = & \sum_{j=1}^{m}I\left((y_{j}\frac{1}{n}\sum_{i=1}^{n}x_{ij})\neq 0\right)\\ & = & \sum_{j=1}^{m}I\left(y_{j}{\left[c\left(x_{1},\ldots ,x_{n}\right)\right]}_{j}\neq 0\right)\\ & = & s(c\left(x_{1},\ldots ,x_{n}\right),y). \end{array}$$

Note that the summations over the instances *i* and dimensions *j* can be inter-changed without affecting the number of non-zero element count because all elements in the feature vectors are non-negative. If this was not the case, we would have zero elements in the centroid because positive and a negative values can potentially cancel out during the computation of the centroid vector.

Several observations can be made about the above-mentioned dimensionality reduction process. First, the basis vectors used for the projection (i.e. prototype vectors) are selected from the given set of feature vectors. This is analogous to the cluster center selection process used in clustering algorithms such as the *k*-medoid clustering [44]. Unlike *k*-means clustering, where the cluster centers are not necessarily data points in the dataset, the *k*-medoid clustering algorithm always selects a data point from the given dataset closest to the centroid of the cluster. Compared to the *k*-means clustering, the *k*-medoid clustering algorithm is less prone to noise (outliers) because of its cluster center selection criterion.

Second, the feature values of the projected vectors are guaranteed to be non-negative because we are considering the inner product between non-negative vectors (recall that character n-gram features are binary valued and contextual features are non-negative real values because we are using PPMI as the co-occurrence weighting measure). This property is particularly useful when we learn cross-lingual projections in the the next Section. Although there are other non-negative dimensionality reduction methods such as non-negative matrix factorization [27], they require optimizing a non-convex objective function, which is both computationally demanding as well as sensitive to the initial conditions. Note that dimensionality reduction methods such as singular value decomposition (SVD) or principal component analysis (PCA) do not necessarily produce non-negative projections even when the feature vectors are non-negative. On the other hand, PVP does not suffer from those drawbacks which makes it an ideal candidate for performing dimensionality reduction for learning a cross-lingual projection. Indeed as we show later, PVP outperforms SVD and NMF on a nearest neighbour prediction task using synthetic sparse data, and cross-lingual biomedical term translation prediction task using four real-world datasets for biomedical terms covering different target languages.

### Learning Cross-Lingual Projections

Once we have represented a term in a particular language using a vector that lists numerous character n-gram features or contextual features extracted from that language, and projected those feature vectors to a non-negative lower-dimensional space using PVP, we learn a cross-lingual projection from the source language to the target language. Ideally, words that are semantically similar in the two languages must be embedded closer to each other in the lower-dimensional embedding.

Consider a pair of terms (*w*_𝓢_, *w*_𝓣_), where *w*_𝓣_ is the translation of *w*_𝓢_. Let us denote the source and target language feature vectors corresponding to *w*_𝓢_ and *w*_𝓣_ respectively by the boldfaced fonts ***w***_**𝓢**_ and ***w***_**𝓣**_. Moreover, let us denote the lower-dimensional projections of ***w***_**𝓢**_ and ***w***_**𝓣**_ respectively by ${\tilde{\text{w}}}_{𝓢}$ and ${\tilde{\text{w}}}_{𝓣}$. Note that the dimensionality of ***w***_**𝓢**_ and ***w***_**𝓣**_ need not be equal, and we may select different numbers of features to represent terms in the source and the target languages. Moreover, the number of prototype vectors selected in the PVP step need not be equal for the source and target languages. For example, we might decide to use 1000 prototype vectors from the source language feature vectors to project source language feature vectors to a 1000 dimensional space, whereas we might select 2000 prototype vectors from the target language feature vectors to project target language feature vectors to a 2000 dimensional space. Therefore, the dimensionalities of ${\tilde{\text{w}}}_{𝓢}$ and ${\tilde{\text{w}}}_{𝓣}$ need not be equal. Given a set of *N* translation pairs ${\left\{({\tilde{w}}_{𝓢}^{\left(i\right)},{\tilde{w}}_{𝓣}^{\left(i\right)})\right\}}_{i=1}^{N}$, we learn a multivariate regression model, *M*, to predict the corresponding target language feature vector ${\tilde{\text{w}}}_{𝓣}$, given its source language feature vector ${\tilde{\text{w}}}_{𝓢}$. Existing bilingual term lexicons or a manually annotated small seed translation term pairs can be used as the train data. We denote the predicted target language feature vector by the learnt multivariate regression model *M* by $M({\tilde{\text{w}}}_{𝓢})$. Because *M* defines a mapping between the source and the target language feature spaces, we call it a **cross-lingual mapping** in the remainder of this paper.

We use Partial Least Squares Regression (PLSR) [4] to learn a regression model using pairs of vectors. PLSR has been applied in Chemometrics [45], producing stable prediction models even when the number of samples is considerably smaller than the dimensionality of the feature space. Given the rank *r* for the regression space, PLSR attempts to project both source and the target language feature vectors to a common *r* dimensional space such that the Pearson correlation coefficient between the two projected vectors is maximized in the lower dimensional space. The rank *r*, is often much smaller than the dimensionalities of the source or target language feature spaces and in practice set to values in the range [10, 100].

Let **X** and **Y** denote matrices formed by arranging respectively the vectors ${\tilde{\text{w}}}_{𝓢}^{\left(i\right)}\text{s}$ and ${\tilde{\text{w}}}_{𝓣}^{\left(i\right)}$ in rows. PLSR decomposes **X** and **Y** into a series of products between rank 1 matrices as follows:

$$\begin{array}{c} X\approx \sum_{l=1}^{r}\lambda_{l}p_{l}^{⊤}=\Lambda P^{⊤} \end{array}$$ (2)

$$\begin{array}{c} Y\approx \sum_{l=1}^{r}\gamma_{l}q_{l}^{⊤}=\Gamma Q^{⊤}. \end{array}$$ (3)

Here, **λ**_*l*_, **γ**_*l*_, ***p***_*l*_, and ***q***_*l*_ are column vectors, and the summation is taken over the rank 1 matrices that result from the outer product of those vectors. The matrices, **Λ**, **Γ**, **P**, and **Q** are constructed respectively by arranging **λ**_*l*_, **γ**_*l*_, ***p***_*l*_, and ***q***_*l*_ vectors as columns.

Pseudo code for learning a cross-lingual mapping, *M*, using PLSR is shown in Table 2 Algorithm 2. It is based on the two block NIPALS routine [46, 47], and iteratively discovers *L* pairs of vectors (**λ**_*l*_,**γ**_*l*_) such that the covariances, Cov(**λ**_*l*_,**γ**_*l*_), are maximised under the constraint ∣∣***p***_*l*_∣∣_2_ = ∣∣***q***_*l*_∣∣_2_ = 1. Finally, the mapping matrix, **M** is computed using **λ**_*l*_,**γ**_*l*_,***p***_*l*_,***q***_*l*_. The predicted vector *M*(***w***_**𝓢**_) of a term *w*_𝓢_ in the source language to the target language is given by

$$\begin{array}{c} M\left(w_{𝓢}\right)=Mw_{𝓢}. \end{array}$$ (4)

**Table 2:** **Algorithm 2** Learning a Cross-Lingual Mapping.

**Input**: **X**, **Y**, Rank *r*.
**Output**: Mapping matrix **M**.
1: Randomly select **γ**_*l*_ from columns in **Y**_*l*_.
2: $v_{l}=X_{l}^{⊤}\gamma_{l}/||X_{l}^{⊤}\gamma_{l}||$
3: **λ**_*l*_ = **X**_*l*_ ***v***_*l*_
4: $q_{l}=Y_{l}^{⊤}\lambda_{l}/||Y_{l}^{⊤}\lambda_{l}||$
5: **γ**_*l*_ = **Y**_*l*_ ***q***_*l*_
6: If **γ**_*l*_ is unchanged go to Line 7; otherwise go to Line 2
7: $c_{l}={\text{λ}}_{l}^{⊤}{\text{γ}}_{l}/||{\text{λ}}_{l}^{⊤}{\text{γ}}_{l}||$
8: $p_{l}={\text{X}}_{l}^{⊤}{\text{λ}}_{l}/{\text{λ}}_{l}^{⊤}{\text{λ}}_{l}$
9: ${\text{X}}_{l+1}={\text{X}}_{l}-{\text{λ}}_{l}{\text{p}}_{l}^{⊤}$ and ${\text{Y}}_{l+1}={\text{Y}}_{l}-c_{l}{\text{λ}}_{l}{\text{q}}_{l}^{⊤}$.
10: Stop if *l* = *r*; otherwise *l* = *l* + 1 and return to Line 1.
11: Let **C** = diag(*c*_1_, …, *c*_*L*_), and **V** = [***v***_1_…***v***_*L*_]
12: **M** = **V**(**P** ^⊤^ **V**)^−1^ **C** **Q** ^⊤^
13: **return M**

### Measuring Cross-Lingual Similarity

Once a cross-lingual mapping is learnt following the steps described in the previous section, we can use it to train a binary random forest classifier to detect translation pairs. Specifically, given a pair (*w*_𝓢_, *w*_𝓣_) of source and target terms that are in a translational relationship, we represent this pair using a feature vector by concatenating the three vectors: (a) ***w***_**𝓢**_, source language feature vector of the source term, (b) ***w***_**𝓣**_, target language feature vector of the target term, and (c) **M** ***w***_**𝓢**_, the projected source language feature vector using the learnt cross-lingual projection, **M**. Let us denote the concatenated vector by [***w***_**𝓢**_;***w***_**𝓣**_;**M** ***w***_**𝓢**_]. Concatenated vectors from correct translation pairs are labeled as positive training instances, whereas we randomly pair a source language term with a target language term to create an equal number of negative training instances. Next, we train a binary random forest model to classify the positive (translational pairs) and negative (non-translational pairs) instances. During test time, we use the class conditional probability returned by the trained random forest model that indicates how likely a given pair of terms belong to the positive class (i.e. correct translations). This probability is considered as the cross-lingual similarity between the source and the target terms. Finally, we rank target language terms in the descending order of the their cross-lingual similarities, and present the top-*k* terms as translation candidates for *w*_𝓢_ to the user. The user (e.g. a human annotator) can then select the best translation for *w*_𝓢_ from the returned ranked list instead of going through a large list of possibly irrelevant terms.

There are several benefits of the above classification-based approach over directly measuring the similarity between a source term and a target term using their feature representations. First, the feature spaces in the source and target languages will not have much overlap, resulting in zero similarity scores.

Second, even if we use the learnt cross-lingual projection model, *M*, to first project the source language feature vector ***w***_**𝓢**_ and then measure the similarity between **M** ***w***_**𝓢**_ and ***w***_**𝓣**_ using some similarity measure such as the cosine similarity, we do not know which common features are salient for computing cross-lingual similarity. Different features might contribute differently to the similarity computation and we would like to learn some weight for each common feature that indicates its importance for detecting cross-lingual similarity. The random forest binary classifier that we train will learn weights representing the discriminative capability of a feature for detecting correct translational term pairs from the incorrect ones.

Third, we might want to consider non-linear combinations of both source and target feature vectors for detecting correct translations. The random forest classifier generates a series of decision trees (commonly known as a forest) that capture numerous combinations of features from both source and target feature vectors. Therefore, by using a random forest classifier instead a linear classifier, we can easily take into account the interaction between source language features and target language features. Indeed, in our preliminary experiments using logistic regression, a linear classification algorithm, resulted in poor performance, showing the importance of considering combinations of features from both source and target languages in order to accurately detect cross-lingual similarities.

## Experiments

We conduct two types of experiments to evaluate the performance of the methods proposed in this paper. First, we evaluate the performance of the PVP method using synthetic data. Second, we evaluate the performance of the proposed cross-lingual similarity measure by using it to detect translations for English source biomedical terms in four target languages: French, Spanish, Greek, and Japanese.

### Evaluating Prototype Vector Projection

The proposed PVP method computes a non-negative lower-dimensional embedding for a given set of feature vectors. Although the absolute similarity scores among vectors in the original (prior to embedding) and the embedded spaces can be different, if the relative ordering of similarity scores are preserved in the embedded space, we can consider such an embedding as desirable for finding nearest neighbors. We use this property to evaluate the performance of a dimensionality reduction method. To explain this idea concretely, let us consider four vectors ***x***,***y***_1_,***y***_2_, and ***y***_3_ in an *m*-dimensional space. Let us denote their projection to a lower *d*(< *m*) dimensional space respectively by $\tilde{\text{x}},{\tilde{\text{y}}}_{1},{\tilde{\text{y}}}_{2}$, and ${\tilde{\text{y}}}_{3}$. Let us assume that the similarity scores, computed using some similarity measure such as cosine similarity, induce the following total ordering sim(***x***,***y***_1_) > sim(***x***,***y***_2_) > sim(***x***,***y***_3_). We can evaluate the accuracy of a dimensionality reduction method by how well this original ordering of neighbors is preserved in the embedded space. Specifically, we can use the same similarity measure to compute the similarity scores $\text{sim}(\tilde{\text{x}},{\tilde{\text{y}}}_{1})$, $\text{sim}(\tilde{\text{x}},{\tilde{\text{y}}}_{2})$, and $\text{sim}(\tilde{\text{x}},{\tilde{\text{y}}}_{3})$ to create a total ordering of the neighbors with respective to $\tilde{\text{x}}$, and compare the original ordering (i.e. *y*_1_ ≻ *y*_2_ ≻ *y*_3_) against the ordering of neighbors with respect to $\tilde{\text{x}}$ after the projection.

Two popular correlation coefficients that have been used for evaluation tasks in natural language processing [48, 49] are the Pearson correlation coefficient (Pearson’s *r*), and the Kendall rank correlation coefficient (Kendall’s *τ*). Pearson’s *r* compares the absolute similarity scores between two vectors before and after the projection, whereas the Kendall’s *τ* ignores the absolute values of similarity and compares only the relative ranking of the neighbors before and after the projection. Therefore, by using both *r* and *τ* as evaluation measures, we can evaluate a dimensionality reduction method for its ability to preserve the topology of a vector space in the embedded space.

To define the evaluation measures we use, let us denote the similarity scores before and after the projection of the neighbors ***y***_*i*_ of a particular vector ***x***_*j*_ respectively by sim(***x***_*j*_,***y***_*i*_) and $\text{sim}({\tilde{\text{x}}}_{j},{\tilde{\text{y}}}_{i})$. Then, the Pearson correlation coefficient computed for the neighbors of ***x***_*j*_, *r*_*j*_, is defined by

$$\begin{array}{c} r_{j}=\frac{\sum_{i=1}^{n-1}\left(\mathrm{sim}\left(x_{j},y_{i}\right)-\mu_{j}\right)\left(\mathrm{sim}\left({\tilde{x}}_{j},{\tilde{y}}_{i}\right)-{\tilde{\mu }}_{j}\right)}{\sqrt{\sum_{i=1}^{n-1}{\left(\mathrm{sim}\left(x_{j},y_{i}\right)-\mu_{j}\right)}^{2}}\sqrt{\sum_{i=1}^{n-1}{\left(\mathrm{sim}\left({\tilde{x}}_{j},{\tilde{y}}_{i}\right)-{\tilde{\mu }}_{j}\right)}^{2}}}, \end{array}$$ (5)

where *μ*_*j*_ and ${\tilde{\mu }}_{j}$ are the sample means of the the similarity scores respectively in the original and the projected spaces. Specifically, they are given by,

$$\begin{array}{ccc} \mu_{j} & = & \frac{1}{n-1}\sum_{i=1}^{n-1}\mathrm{sim}\left(x_{j},y_{i}\right),\\ {\tilde{\mu }}_{j} & = & \frac{1}{n-1}\sum_{i=1}^{n-1}\mathrm{sim}\left({\tilde{x}}_{j},{\tilde{y}}_{i}\right). \end{array}$$

Note that because there are *n* vectors in the dataset, the total number of neighbors for each vector is the remainder of (*n*−1) vectors.

The Kendall’s *τ* for the same two groups of similarity scores are defined as follows

$$\begin{array}{c} \tau_{j}=\frac{4C(\phi_{j},\sigma_{j})}{\left(n-1)(n-2\right)}-1. \end{array}$$ (6)

Here, *ϕ*_*j*_ and *σ*_*j*_ denote the total orderings of indices of the neighbors of *x*_*j*_ respectively in the original and the embedded spaces, sorted in the descending order of their similarity scores with respect to ***x***_*j*_ and ${\tilde{\text{x}}}_{j}$, and *C*(*ϕ*_*j*_, *σ*_*j*_) is the number of concordant pairs between the two permutations. A pair of elements (*p*, *q*) is said to be concordant if the ranks assigned to *p* and *q* in the two orderings do not contradict (i.e. if *p* ≻ *q* in both *σ* and *ϕ*, or *p* ≺ *q* in both *σ* and *ϕ*).

We compute the Pearson’s *r* and Kendall’s *τ* coefficients for the dataset consisting of *N* instances as the average over the individual coefficients as follows

$$\begin{array}{ccc} r & = & \frac{1}{n}\sum_{j=1}^{n}r_{j},\\ \tau & = & \frac{1}{n}\sum_{j=1}^{n}\tau_{j}. \end{array}$$

Both Pearson’s *r* and Kendall’s *τ* coefficients are in the range [−1,1], where higher values indicate positive correlations between the similarity scores (or relative rankings in the case of Kendall’s *τ*) in the original and the embedded spaces. Among different dimensionality reduction methods that project the same set of *n* dimensional vectors to the same *d* lower-dimensional space, we prefer methods that produce high Pearson’s *r* and Kendall’s *τ* values.

#### Dimensionality Reduction Performance using Synthetic Data

To compare the accuracy of the proposed PVP method against relevant dimensionality reduction methods we generate two synthetic datasets. For the first synthetic dataset, we generate 1000 random vectors each with 1000 dimensions. All the elements in the vectors are non-negative and uniformly selected from the interval [0, 1]. In natural language processing, it is common to have highly sparse feature vectors. Although the set of features is huge, only a handful of features appear in any single training instance such as a document. For example, both the character n-gram feature vectors and contextual feature vectors in our task are very sparse. Ideally, the synthetic data must capture this phenomenon faithfully as possible so that any conclusions that are drawn using the synthetic datasets can be generalized to NLP tasks that require some form of a dimensionality reduction step. To reflect this phenomenon in the synthetic data, we randomly set elements in our feature vectors to zero with a 0.98 probability. Specifically, for each element in each feature vector we generate, we draw a random sample uniformly from the interval [0, 1], and set the corresponding element to zero if the drawn sample is less than 0.98. By following this process we obtain feature vectors that have on average 98% zeros. Following the same procedure, we create another dataset that has 1000 random vectors each with 10,000 dimensions. By using two synthetic datasets that have feature vectors of different dimensionalities we are able to verify whether the trends observed depend on the dimensionality of the feature vectors.

We compare the Pearson and Kendall correlation coefficients obtained under varying dimensionalities for the proposed prototype vector prediction (**PVP**) method against several dimensionality reduction methods as described next.

- **L2** We select the *d* feature vectors with the largest L2 norms as prototype vectors. The L2 norm of an *n*-dimensional vector ***x*** is defined as $\sqrt{\sum_{j=1}^{n}{x_{i}}^{2}}$. Recall that in Table 1 Algorithm 1, PVP selects prototype vectors based on the number of non-zero elements in feature vectors. An alternative approach would be to use L2 norm because if most of the elements in a vector are non-zero, it will result in a high L2 norm. However, this property does not always hold. For example, we might have feature vectors that contain only a handful of non-zero features but their absolute values might be large, resulting in high L2 norms. Nevertheless, feature vectors that are dense are likely to yield non-zero values when the cosine similarities are computed using them, which is useful when computing embeddings for the feature vectors. Note that L1 norm, given by $\sum_{j=1}^{n}|x_{j}|$, can also be used as a baseline method for selecting prototype vectors. However, both L1 and L2 norms induce the same total ordering among a given set of feature vectors. Therefore, we would obtain the same set of prototype vectors using both L1 and L2 norms. As a general result, it can be proved that any two norms ∣∣ ⋅ ∣∣_*α*_ and ∣∣ ⋅ ∣∣_*β*_ in a finite *d* dimensional vector space are equivalent in the sense that there exist *m*, *M* ∈ ℝ such that for a vector ***x*** ∈ ℝ^*d*^ the inequalities *m*∣∣***x***∣∣_*α*_ ≤ ∣∣***x***∣∣_*β*_ ≤ *M*∣∣***x***∣∣_*α*_ hold [41]. Therefore, equivalent total orderings will be induced by different norms. We use L2 norm as a baseline method in our experiments to demonstrate the level of performance we would obtain if we simply use the vector norm for selecting prototype vectors.
- **SVD**Singular value decomposition (SVD) is a popular technique in NLP for performing dimensionality reduction. It has been used in both attributional and relational similarity measurement tasks to overcome the sparseness of feature vectors, thereby reducing zero similarity scores [50]. Given a matrix **A** (not necessarily a square matrix), SVD decomposes **A** into the product of three matrices **U**, **D**, and **V** given by
  $$\begin{array}{c} A=UDV^{⊤}, \end{array}$$
  where **U** and **V** are in column orthonormal form (i.e. **U** ^⊤^ **U** = **V** ^⊤^ **V** = **I**), and **D** is a diagonal matrix containing the singular values of **A** as the diagonal elements [41]. The rank of **D** is equal to that of **A**. For an integer *d* lesser than the rank of **A**, the matrix $\hat{\text{A}}={\text{U}}_{d}{\text{D}}_{d}{\text{V}}_{d}^{⊤}$ gives the *d*-dimensional approximation to **A** in the sense that the Frobenius norm of the approximation error, $||\text{A}-\hat{\text{A}}|{|}_{F}$ is minimized by $\hat{\text{A}}={\text{U}}_{d}{\text{D}}_{d}{\text{V}}_{d}^{⊤}$ among all matrices with rank *d*. Here, **U**_*d*_ and **V**_*d*_ are created by selecting respectively the left and right singular vectors of **U** and **V** corresponding to the largest singular values of **A**[40]. However, the elements in **A**_*d*_ are not necessarily non-negative even if the elements in **A** are non-negative. This becomes problematic when computing cosine similarity scores giving rise to negative similarity scores. In practice, approximately half of the elements in **A**_*d*_ are negative. Turney [51] proposed a solution to this problem by setting the negative values in **A**_*d*_ to zero. We implement this method as a baseline for comparison.
- **NMF** Given a matrix **A** ∈ ℝ^*n* × *m*^ that contains non-negative elements, non-negative matrix factorization (NMF) [27, 52, 53] decomposes **A** into the product of two non-negative matrices **W** ∈ ℝ^*n* × *k*^ and **H** ∈ ℝ^*k* × *m*^ such that,
  $$\begin{array}{c} A=WH. \end{array}$$
  In particular, the matrix **W** can be seen as a lower *k*-dimensional representation of the given *n* vectors. We use the rows in matrix **W** computed using projected gradient-based NMF algorithm implemented in scikit-learn library (http://scikit-learn.org/) as a baseline dimensionality reduction method for comparisons.

**Fig 1:**
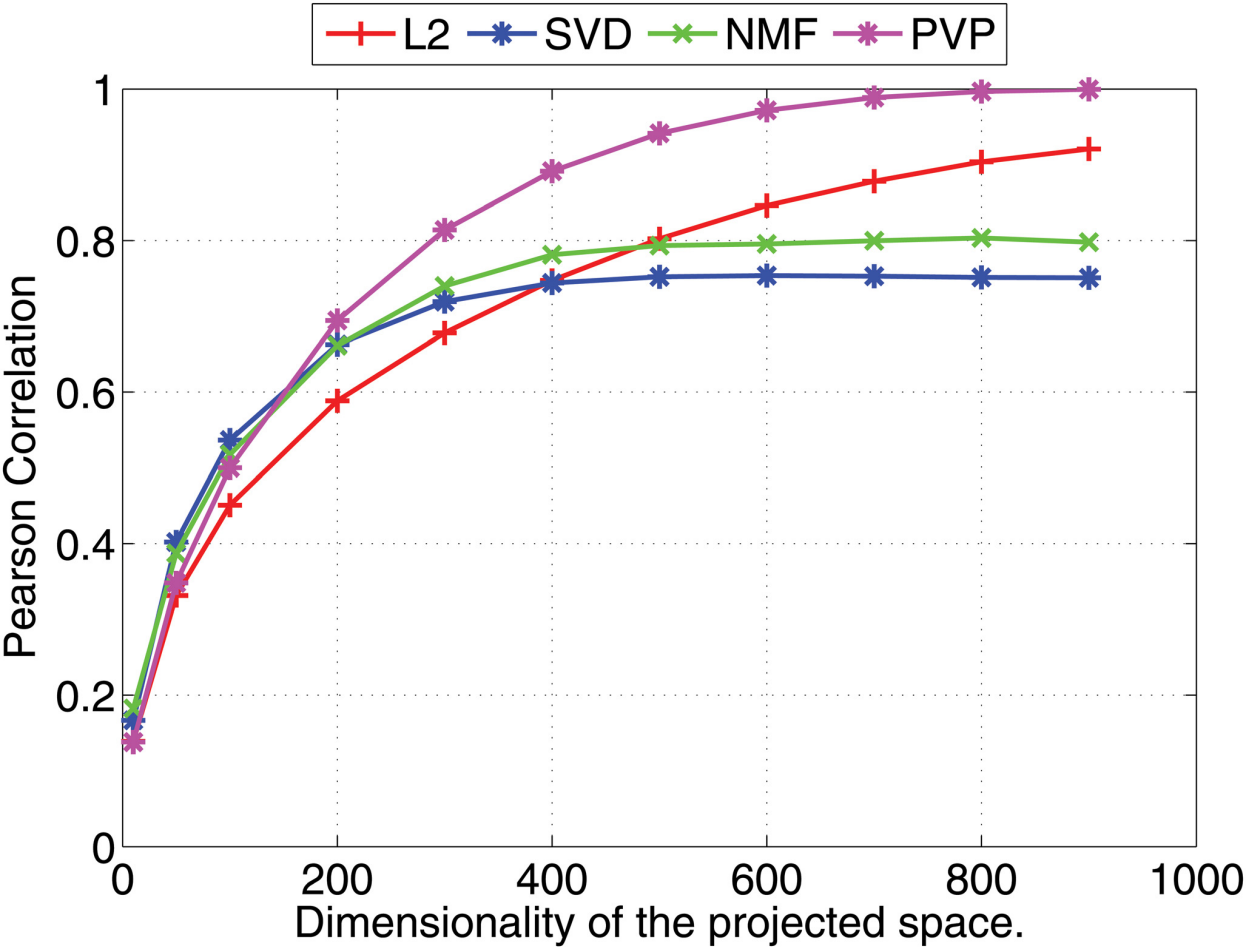
Nearest neighbor prediction with artificial data. Pearson’s *r* correlation coefficients for different dimensionality reduction methods are shown under varying dimensionalities (Feature vectors are 1000 dimensional).

**Fig 2:**
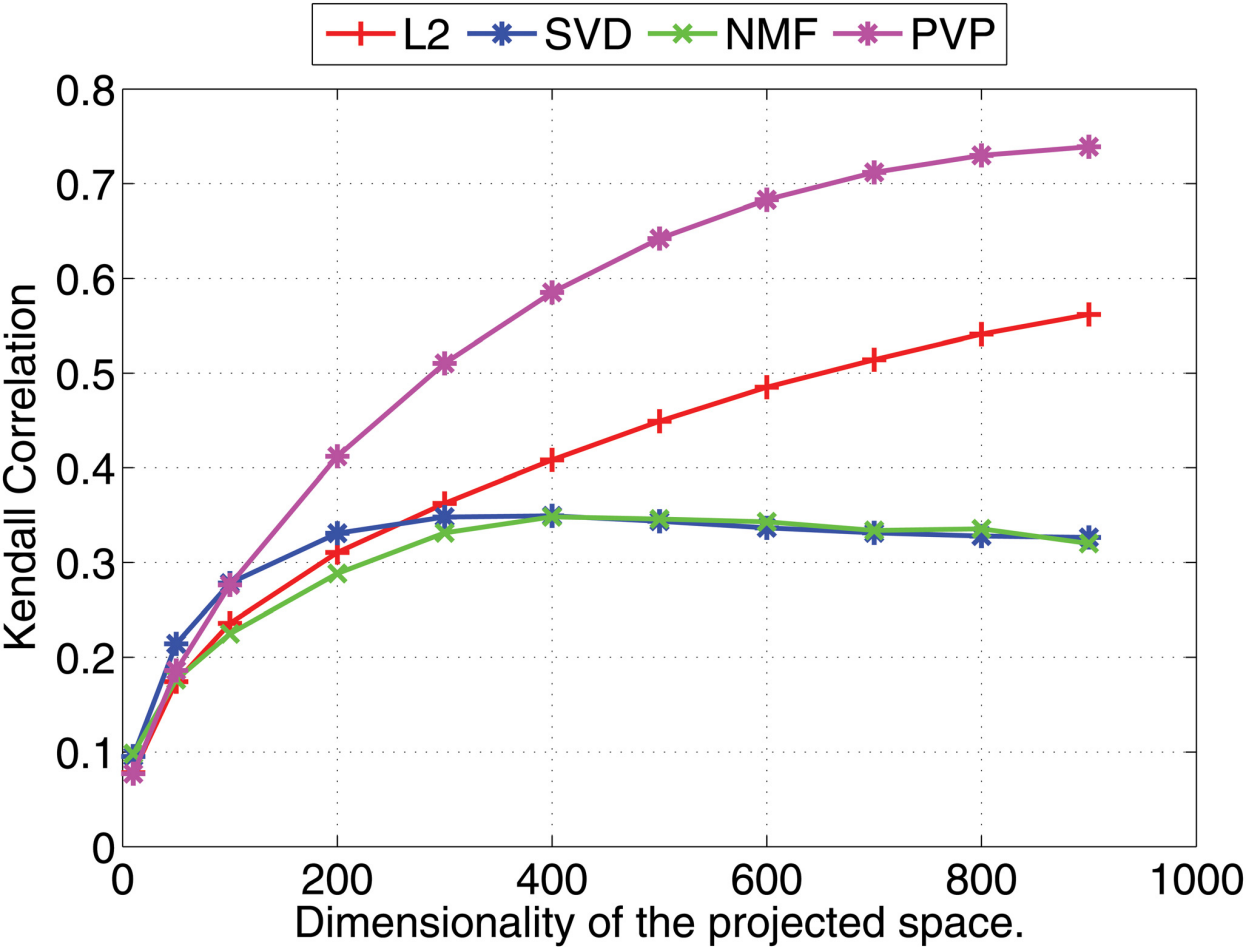
Nearest neighbor prediction with artificial data. Kendall’s *τ* (right) correlation coefficients for different dimensionality reduction methods are shown under varying dimensionalities (Feature vectors are 1000 dimensional).

**Fig 3:**
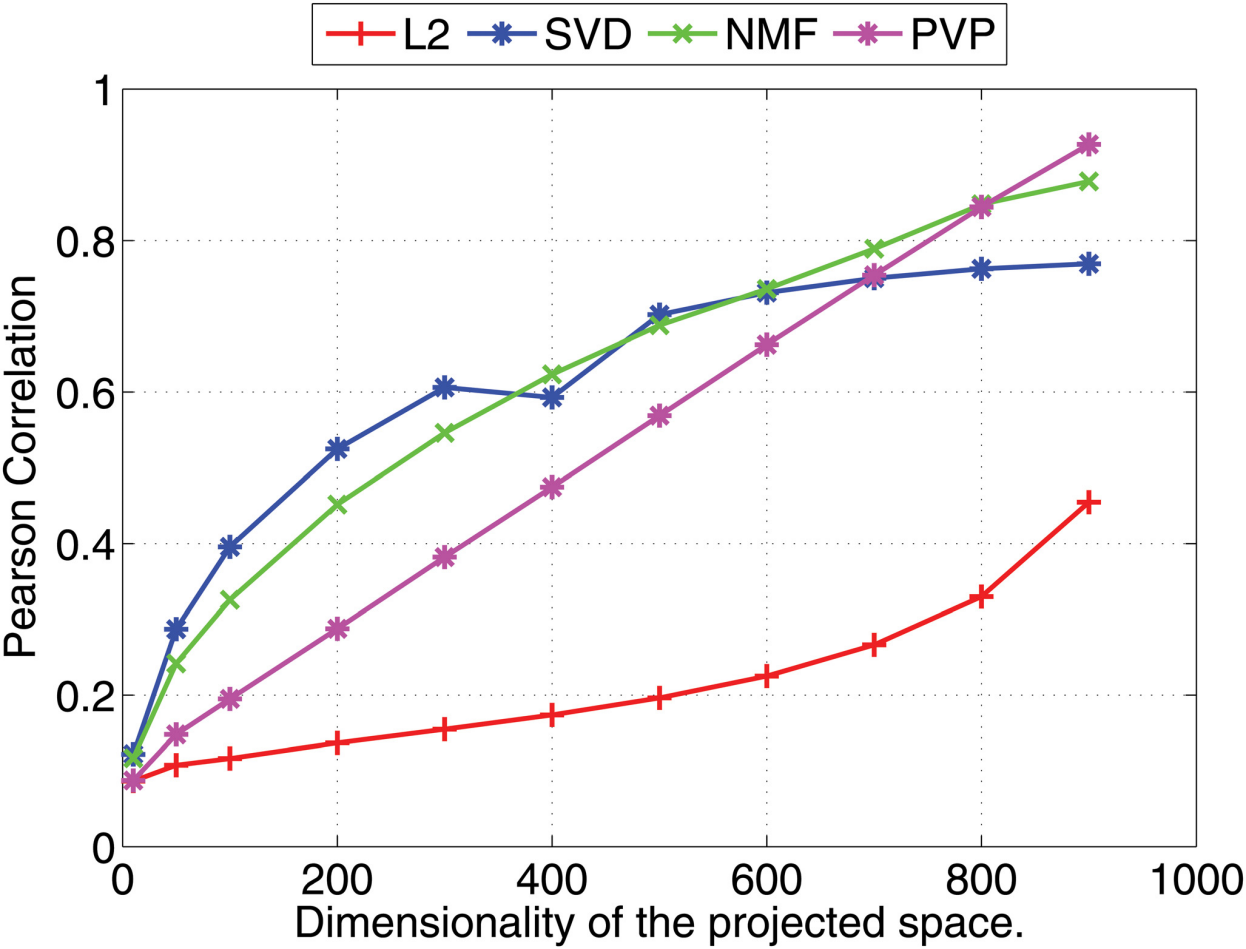
Nearest neighbor prediction with artificial data. Pearson’s *r* correlation coefficients for different dimensionality reduction methods are shown under varying dimensionalities (Feature vectors are 10000 dimensional).

**Fig 4:**
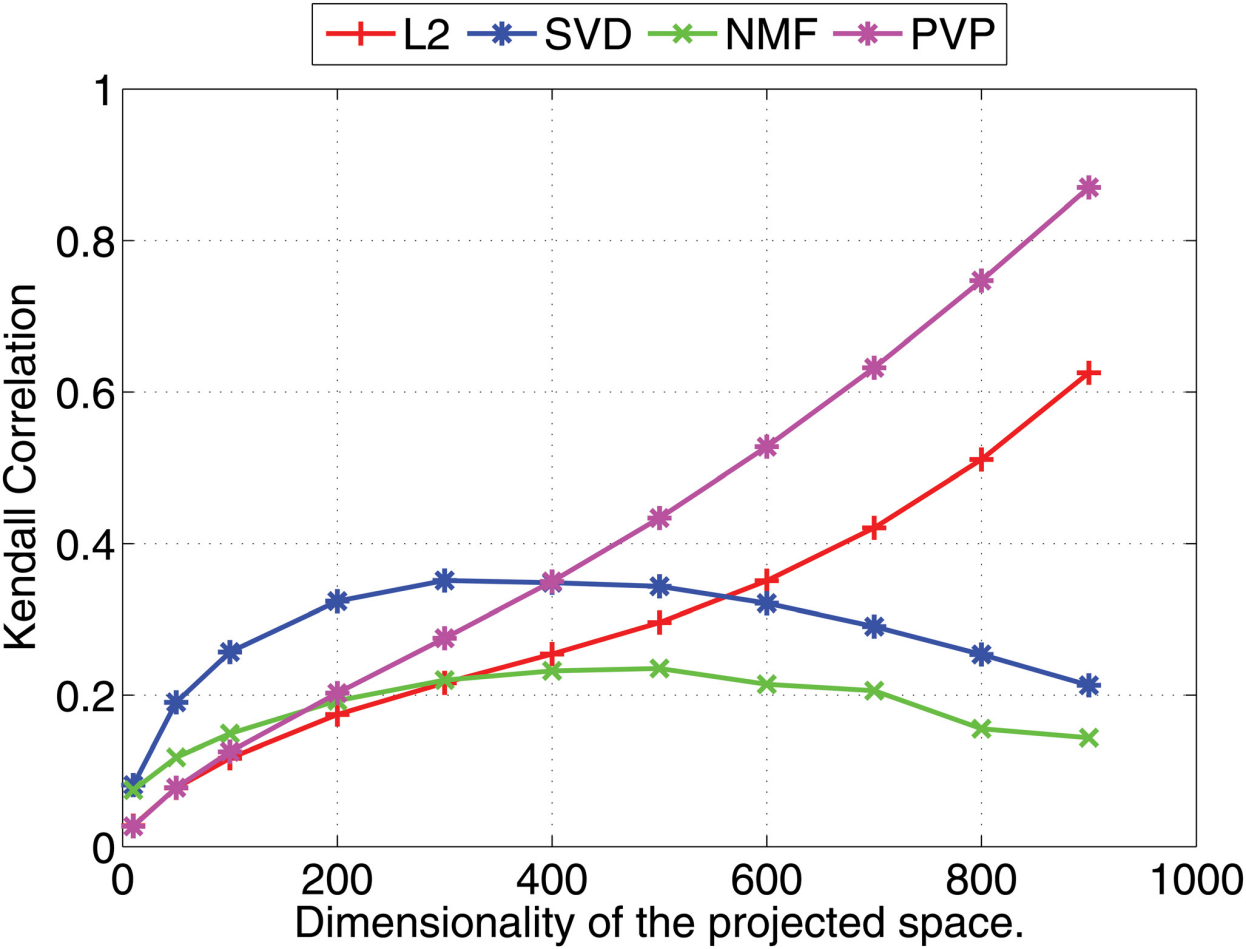
Nearest neighbor prediction with artificial data. Kendall’s *τ* (right) correlation coefficients for different dimensionality reduction methods are shown under varying dimensionalities (Feature vectors are 10000 dimensional).

Experimental results are shown in Figs 1 and 2 (for 1000 dimensional feature vectors) and Figs 3 and 4 (for 10,000 dimensional feature vectors). As an overall trend in both Figures, we see that all methods are performing equally when the dimensionality of the projected space is very small. However, the correlation coefficients for such low dimensional projections are also very low because most of the important features of the original space are lost as a result of the aggressive lower dimensional projections. When we increase the dimensionality both Pearson and Kendall correlation coefficients improve. However, **SVD** and **NMF** methods quickly saturates to almost fixed correlations and by further increasing the dimensionality we cannot improve their performance. On the other hand, the correlation coefficients with **PVP** continuously increase. Because **SVD** and **NMF** are computing low rank approximations to the matrix defined by the feature vectors, the correlation does not improve when we have reached the rank of the data matrix. Moreover, minimization of the Frobenius norm of the approximation as done by **SVD** does not guarantee a high correlation between similarity scores computed using the lower dimensional projections of the feature vectors. In the larger 10,000 dimensional setting depicted in Figs 3 and 4, we see that Kendall’s *τ* drops for **SVD** and **NMF** methods when the dimensionality is increased beyond 300 dimensions. In practice, it is difficult to determine the optimal value of the dimensionality for the projection. Therefore, in practice projection methods that do not loose performance due to extra dimensions are desirable. Performance of the **L2** baseline varies and is not robust. For example, in the 10,000 dimensional case (Fig 3), **L2** method reports the worst Pearson correlation among the four methods compared.

Overall, the projected feature vectors using **NMF** are almost 100% dense, whereas **SVD** with negative values set to zero produces ca. 50% dense vectors. Vectors projected by **PVP** are ca. 60% dense, giving an intermediate level of density compared to **NMF** and **SVD**. In particular, considering that the original feature vectors were only 2% dense (i.e. 98% sparse), all three methods can be seen as producing dense vectors in the lower-dimensional space. Although we do not explore data visualization in this paper, the different densities produced by these methods could be of interest to lower-dimensional data visualization tasks [54].

**Fig 5:**
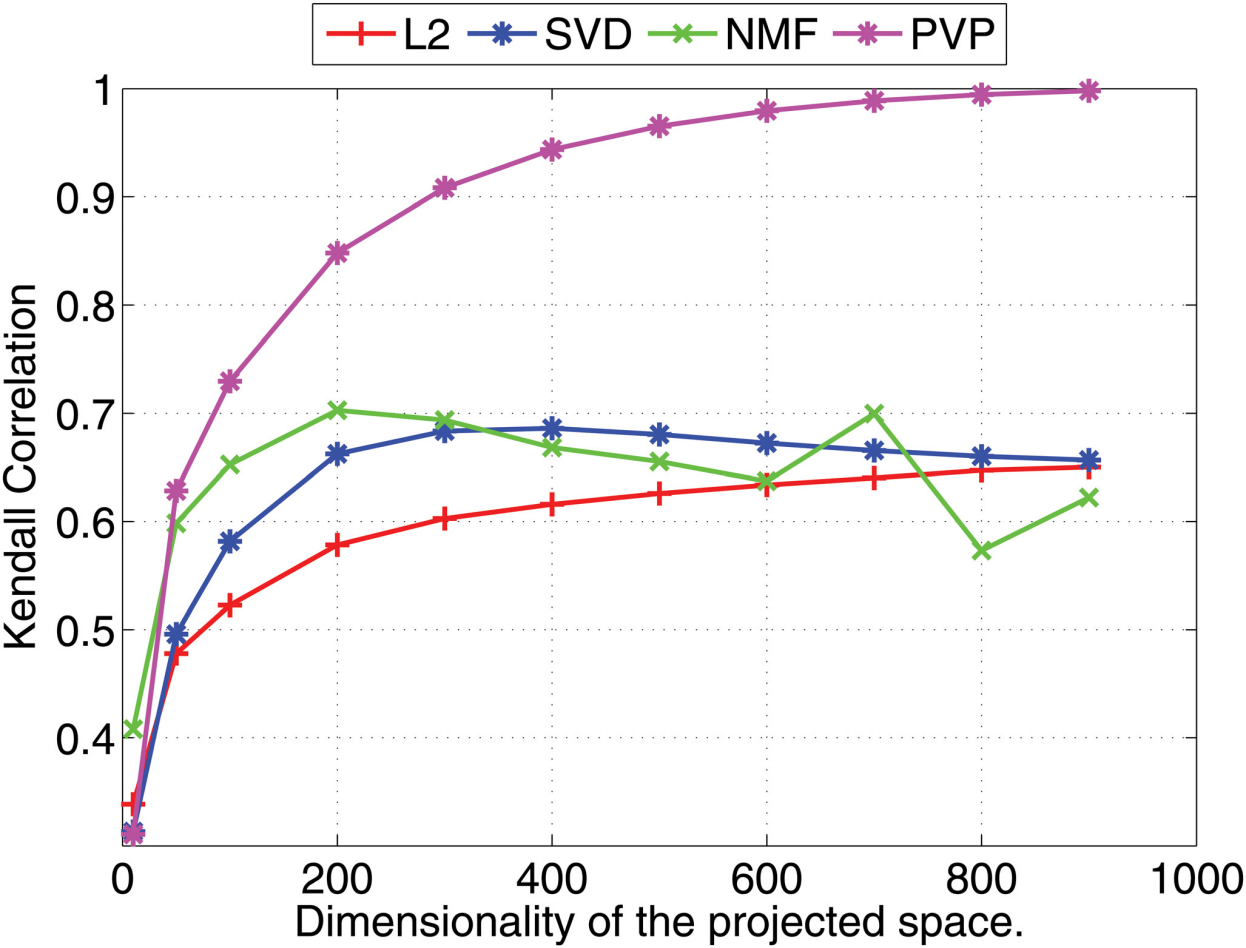
Nearest neighbor prediction with English feature vectors. Pearson’s *r* correlation coefficients for different dimensionality reduction methods are shown under varying dimensionalities (Feature vectors are 1000 dimensional).

**Fig 6:**
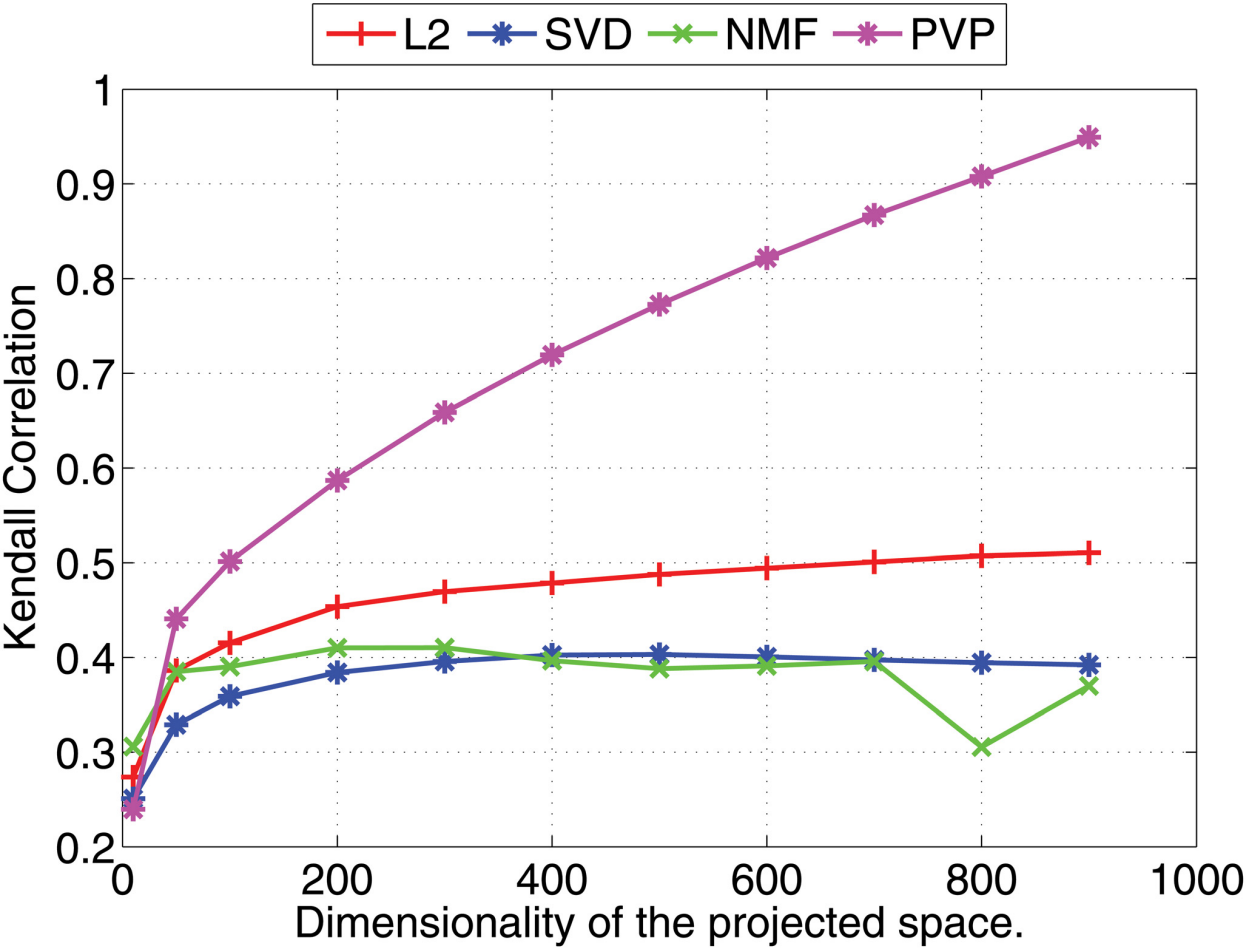
Nearest neighbor prediction with English feature vectors. Kendall’s *τ* (right) correlation coefficients for different dimensionality reduction methods are shown under varying dimensionalities (Feature vectors are 1000 dimensional).

In addition to the experiments described above which use artificial data, we also conduct a second experiment using the monolingual feature vectors we created for the English source language. We use 1000 dimensional contextual feature vectors for 2454 English source terms, and apply the dimensionality reduction methods described in the previous paragraph. We compare the nearest neighbors in the original feature space to that in the projected feature space using Kendall rank correlation coefficient and the Pearson’s correlation coefficient as shown in Figs 5 and 6. Similar trends that were observed in the experiments conducted using artificial data can be observed in Figs 5 and 6, and the difference between PVP and other methods is more significant.

### Evaluating Cross-Lingual Similarity Measurement

#### Cross-Lingual Biomedical Terms Dataset

To evaluate the proposed cross-lingual similarity measure, we apply it in a biomedical term translation detection task. We use the dataset created by Kontonatsios et al. [24], which lists translations for English biomedical terms in four target languages: French, Spanish, Greek, and Japanese. Next, we briefly describe the process followed by Kontonatsios et al. [24] to construct this dataset. First, 4000 English Wikipedia articles are selected covering 4000 English biomedical terms. Second, Wikipedia interlingual links are used to retrieve thematically related articles in each of the target languages. However, not all of the 4000 English articles are translated into all the target languages. Therefore, different lists of query-terms were used to retrieve Wikipedia articles for different language pairs. For training purposes, we used 5,000 pairs of source and target language terms for each language pair, whereas, for testing additional 1000 source terms were used. Train and test term pairs are manually selected from UMLS.

#### Evaluation Measure

We compute precision@R for different levels of rank R. Precision@R is defined as

$$\begin{array}{c} \mathrm{Precision}@R=\frac{\text{total}\;\text{number}\;\text{of}\;\text{correct}\;\text{translations}\;\text{among}\;\text{top-R}\;\text{ranked}\;\text{candidates}}{R}. \end{array}$$ (7)

We compute Precision@R values for each test case in our test dataset and compute the average of those values as the evaluation measure. Precision@R is a standard evaluation measure in information retrieval [55], where the search results ranked and returned by an information retrieval system is evaluated for its precision at different ranks. An information retrieval system that ranks relevant results among the top ranked candidates is desirable, and Precision@R measure captures this notion.

#### Results

We use Precision@R values to evaluate character n-gram features and contextual features as used in the proposed method. We compare the proposed **PVP**-based dimensionality reduction method against **SVD** and **NMF** methods, which were defined under the experiments conducted using synthetic data. Specifically, in **PVP**, **SVD**, and **NMF** methods, we represent a term pair (*w*_𝓢_, *w*_𝓣_) using the concatenated vector $\left[{\tilde{\text{w}}}_{𝓢};{\tilde{\text{w}}}_{𝓣};\text{M}{\tilde{\text{w}}}_{𝓢}\right]$ when training the random forest classifier. The **No** baseline denotes the level of performance we would obtain if we had not use any dimensionality reduction methods, but had used the original feature spaces to train the random forest binary classifier. Specifically, in **No** baseline, we represent a term pair (*w*_𝓢_, *w*_𝓣_) using the concatenated vector [***w***_**𝓢**_;***w***_**𝓣**_;**M** ***w***_**𝓢**_] when training the random forest classifier, Any differences in the final performance between **No** and each of the **SVD**, **NMF**, and **PVP** methods can be directly attributable to the learnt cross-lingual projection model *M* using each dimensionality reduction method.

We use 10,000 dimensional feature vectors for source and target term representations with both character n-grams as well as contextual features. Prior work studying cross-lingual biomedical term translations has reported that 10,000 features are sufficient to obtain high-quality translation candidates [23]. The proposed PVP method was used to project these 10,000 dimensional feature space to 2,000 dimensions. We learn the PLSR model in Table 2 Algorithm 2 with 10 components. Random forest classifier is trained with 150 trees. Both **SVD** and **NMF** methods are used with 2,000 dimensions, the exact same number of dimensions as used by **PVP**. Therefore, any differences among **PVP**, **SVD**, and **NMF** methods can be considered as resulting from their dimensionality reduction method instead of the dimensions.

For each target language, we denote the level of performance for ranks 1–10. In each test case, we have one or two correct target language translations listed for each source language term. In order to obtain non-zero Precision@R values, a method must rank those correct translations among the top 10 ranked candidates from a pool of 1000 target language terms. We consider this strict evaluation criteria not only enables us to differentiate the performance of the different methods we compare, but also resembles the perceived application of the proposed method—suggesting target language translation candidates for a given source language biomedical term to support the manual annotation process of biomedical bilingual dictionaries.

From Table 3 we see that for French, Greek, and Japanese languages there is a clear benefit of using the projected features produced by the proposed **PVP**. However, the performance decreases when projected features are used for Spanish. For all four target languages, **PVP** consistently outperforms **SVD** and **NMF**, demonstrating its accuracy as a dimensionality reduction method.

**Table 3:** Precision@rank values for English as the source language and different target languages using character n-gram features.

**French**	No	SVD	NMF	PVP
@1	0.263	0.121	0.091	0.297
@2	0.369	0.194	0.163	0.415
@3	0.439	0.254	0.238	0.494
@4	0.506	0.337	0.289	0.539
@5	0.543	0.377	0.345	0.574
@6	0.564	0.415	0.381	0.597
@7	0.590	0.458	0.411	0.615
@8	0.614	0.501	0.470	0.624
@9	0.636	0.536	0.509	0.640
@10	0.647	0.556	0.546	0.653
**Spanish**	No	SVD	NMF	PVP
@1	0.145	0.086	0.112	0.130
@2	0.243	0.150	0.166	0.200
@3	0.304	0.215	0.228	0.261
@4	0.358	0.270	0.263	0.324
@5	0.401	0.319	0.322	0.385
@6	0.443	0.361	0.370	0.420
@7	0.478	0.401	0.412	0.457
@8	0.508	0.435	0.449	0.492
@9	0.549	0.490	0.476	0.521
@10	0.576	0.532	0.517	0.543
**Greek**	No	SVD	NMF	PVP
@1	0.07	0.089	0.101	0.224
@2	0.099	0.170	0.166	0.305
@3	0.136	0.235	0.224	0.351
@4	0.172	0.284	0.284	0.375
@5	0.191	0.331	0.333	0.390
@6	0.211	0.369	0.383	0.403
@7	0.242	0.420	0.426	0.409
@8	0.256	0.456	0.458	0.418
@9	0.274	0.490	0.491	0.426
@10	0.296	0.529	0.528	0.431
**Japanese**	No	SVD	NMF	PVP
@1	0.048	0.041	0.018	0.162
@2	0.066	0.075	0.043	0.193
@3	0.097	0.094	0.060	0.223
@4	0.127	0.115	0.086	0.250
@5	0.142	0.143	0.103	0.261
@6	0.164	0.153	0.126	0.274
@7	0.179	0.168	0.140	0.287
@8	0.204	0.191	0.168	0.297
@9	0.218	0.210	0.191	0.303
@10	0.232	0.224	0.208	0.314

In the case of contextual features shown in Table 4, we see that **PVP** reports the best results for all rank levels for French and Spanish. For Greek and Japanese, **PVP** reports better results when we consider all rank values except at the first rank. Nevertheless, **PVP** consistently outperform **SVD** and **NMF** for all target languages and all ranks when we use contextual features, as it did with the character n-gram features. The ability of the proposed **PVP** method to work well with different types of features is important because it enables us to experiment with numerous feature types. Overall, compared to character n-gram feature-based results shown in Table 3, the Precision@R values obtained using contextual features shown in Table 4 are low. Compared to the character n-gram features, the distribution of the context features is dispersed because of the large number of unigrams and bigrams extracted as contextual features. Therefore, contextual feature vectors are high dimensional and sparse compared to character n-gram feature vectors. Moreover, contextual features are extracted from the local context of the terms and might not necessarily be strongly related to the term under consideration. Therefore, the association measure we use to evaluate the strength of the relationship between a term and its contextual features becomes important. On the other hand, character n-gram features are extracted directly from the term itself, and does not require any weighting.

**Table 4:** Precision@rank values for English as the source language and different target languages using contextual features.

**French**	No	SVD	NMF	PVP
@1	0.083	0.038	0.063	0.090
@2	0.148	0.095	0.111	0.173
@3	0.202	0.141	0.177	0.231
@4	0.248	0.189	0.223	0.275
@5	0.294	0.231	0.261	0.325
@6	0.319	0.266	0.300	0.369
@7	0.347	0.295	0.332	0.402
@8	0.371	0.323	0.361	0.437
@9	0.394	0.352	0.404	0.459
@10	0.420	0.379	0.437	0.478
**Spanish**	No	SVD	NMF	PVP
@1	0.070	0.042	0.087	0.103
@2	0.121	0.094	0.143	0.182
@3	0.185	0.145	0.192	0.245
@4	0.241	0.177	0.230	0.289
@5	0.273	0.217	0.289	0.341
@6	0.317	0.250	0.337	0.389
@7	0.354	0.295	0.382	0.436
@8	0.382	0.332	0.415	0.471
@9	0.419	0.368	0.462	0.498
@10	0.457	0.397	0.487	0.536
**Greek**	No	SVD	NMF	PVP
@1	0.044	0.031	0.038	0.036
@2	0.068	0.063	0.066	0.085
@3	0.103	0.080	0.085	0.119
@4	0.129	0.105	0.110	0.164
@5	0.151	0.125	0.134	0.184
@6	0.178	0.147	0.152	0.225
@7	0.197	0.165	0.170	0.238
@8	0.223	0.191	0.192	0.264
@9	0.240	0.212	0.204	0.285
@10	0.256	0.236	0.219	0.298
**Japanese**	No	SVD	NMF	PVP
@1	0.037	0.018	0.027	0.032
@2	0.056	0.031	0.046	0.064
@3	0.090	0.044	0.057	0.080
@4	0.094	0.057	0.071	0.102
@5	0.108	0.068	0.080	0.126
@6	0.116	0.083	0.109	0.134
@7	0.133	0.093	0.129	0.149
@8	0.144	0.106	0.140	0.159
@9	0.151	0.114	0.153	0.170
@10	0.168	0.126	0.168	0.179

A particularly interesting observation from our analysis is the high precision scores obtained using the character n-gram feature projection in the English-Japanese translation task. These two languages use different alphabets. Therefore, the character n-gram feature spaces between English and Japanese do not overlap. Despite this difficulty, we can learn a cross-lingual projection model between the character n-gram feature spaces between English and Japanese that can be used to correctly identify translations. Character n-gram models have been reported to be sensitive to the distance between languages in prior work on term translation [24].

The dimensionality reduction conducted by PVP can be seen as projecting similar features in each language to the same dimension in the lower-dimensional space. On the other hand, the random forest classifier can be seen as considering the interactions between the dimensions in the source and target language lower-dimensional spaces created by PVP. The cross-lingual projection further improves the cross-lingual similarity measurement by reducing the mismatch between source and target feature spaces.

## Discussion and Conclusions

We considered the problem of measuring the similarity between technical terms such as biomedical terms across different languages. For this purpose, we proposed a cross-lingual similarity measure by first representing source and target language terms using character n-gram features or contextual features. We then project feature vectors in each language to a lower-dimensional space to reduce the number of features used in the representations. We proposed PVP for this purpose. PVP selects a subset of the feature vectors as prototype vectors and use those as basis vector to project the given set of feature vectors to a lower dimensional space.

Next, we proposed a method to learn a cross-lingual projection model using the partial least squares regression (PLSR). Finally, we trained a binary random forest classifier to discriminate positive (translational pairs) vs. negative (non-translational pairs), and use the class conditional probability returned by the trained random forest classifier as a cross-lingual similarity measure to rank the target language translational candidates for a given source language term.

We compared the proposed PVP method against previously proposed dimensionality reduction methods such as the singular value decomposition (SVD), and non-negative matrix factorization (NMF), as well as a baseline method that uses the L2 norm of the feature vectors to select the prototype vectors. Our experimental results on two synthetic datasets show the superiority of the proposed PVP method for dimensionality reduction. We apply the proposed cross-lingual similarity measure to find translations for English source terms in four target languages: French, Spanish, Greek, and Japanese. Our experimental results show that except for Spanish, for all other languages we can improve the performance of the translation detection by projecting character n-gram features.

Several interesting future research directions of the current work can be identified. Given that character n-gram features and contextual features capture different properties of terms (i.e. intrinsic vs. extrinsic), an obvious next step would be to combine those feature spaces to better represent a pair of terms. We note that the main contributions of our work, PVP and cross-lingual projection learning, are independent of any feature spaces that we use to represent terms in the source and the target languages. Therefore, in principle our proposed methods can be used with different feature spaces. Improving cross-lingual similarity measurement using different features is a complementary research direction beyond the scope of this paper. Nevertheless we mention that there are several important challenges that one must overcome when combining different feature spaces such as weighting and normalizing different groups of features in a consistent manner. For example, character n-gram features are binary whereas PPMI-weighted contextual features are real-valued. How to combine these two types of features and whether we should normalize feature vectors prior to training, if so using which norm, are all important design decisions.

A different research direction would be to exploit the compositionality of technical terms when constructing feature vectors to represent the technical terms. For example, a term that has three constituent words might appear only a small number of times even in a large corpus, which makes it difficult to extract sufficiently larger number of contextual features to represent the term. This leads to sparse contextual feature vectors, which becomes problematic during cross-lingual similarity measurement. On the other hand, the individual words in the term might be more popular and are likely to have many contextual features. Therefore, if we can compute the representation for the term using the representations for the individual words in the term, we can obtain a dense representation for the term.

Several variants of the PVP algorithm presented in Table 1 Algorithm 1 are possible depending the structure of the data. For example, if the dataset that we would like to perform dimensionality reduction on consists of multiple clusters, then we could first apply a clustering algorithm such as k-means to produce the inherent clusters in the dataset. Next, we can select a set of prototypes considering each cluster separately to select a representative set of prototype vectors that could be used for the dimensionality reduction step.

The prototype selection criterion of PVP can also be replaced with other criteria. For example, we could select prototype vectors not only by their ability to produce dense vectors in the projected space but also to maximize the diversity of the selected set of prototype vectors. PVP algorithm presented in Table 1 Algorithm 1 selects prototype vectors based on the number of non-zero elements in them, and attempts to reduce the redundancy in the prototype vectors by iteratively removing the prototype vectors selected at each round. Alternatively, we could select prototype vectors that simultaneously maximizes an objective function consisting of two factors: (a) average number of non-zero elements in the set of prototype vectors selected so far, and (b) the average pairwise dissimilarity between all pairs of prototype vectors selected so far.

In our future work, we plan to explore these research directions.

## Supporting Information

S1 dataset We publicly release the processed feature vectors used by the proposed method to facilitate future comparisons. (TGZ)
